# July Phenomenon Impacts Efficiency of Emergency Care

**DOI:** 10.5811/westjem.2018.10.39885

**Published:** 2018-11-19

**Authors:** Amit Bahl, Catherine Cooley Hixson

**Affiliations:** * Beaumont Hospital, Department of Emergency Medicine, Royal Oak, Michigan; † Michigan State University, Department of Emergency Medicine, Grand Rapids Emergency Medical Education Partners, Grand Rapids, Michigan

## Abstract

**Introduction:**

The “July effect” describes the period in which new interns begin learning patient care while senior residents take on additional responsibility in an academic hospital setting. The annual change in staffing creates inefficiencies in patient care, which may negatively impact quality of care. Our objective was to evaluate the impact of the annual resident turnover on emergency department (ED) efficiency in a teaching hospital.

**Methods:**

This was an institutional review board-approved retrospective chart review spanning two academic years analyzing 79,921 records. We grouped July and August into the period of least experience (PLE) and May and June into the period of most experience (PME). Outcomes included faculty and resident productivity, ED door-to-doctor time, and time to disposition.

**Results:**

Patients were evaluated by 117 emergency residents and 73 emergency faculty. We excluded patient records for 35 off-service residents. Residents saw 15.8% more patients in the PME compared to the PLE (p<0.0001). The residents’ average door-to-doctor time during the PLE was 45.63 minutes (standard deviation [SD] 33.01, median 36) compared to 34.69 minutes (SD 25.22, median 28) during the PME, with a decrease in time by 21.3% (p=0.0203). The residents’ average time to disposition during the PLE was 304.6 minutes (SD 308, median 217) compared to 269.0 minutes (SD 282, median 194) during the PME, decreasing by 12.4% (p=0.0001). Residents had an average ED length of stay for discharged patients of 358.5 minutes (SD 374.6, median 238) during the PLE compared to 309.9 minutes (SD 346.4, median 209) during the PME, decreasing 13.7% for discharged patients (p=0.0017).

**Conclusion:**

Annual turnover of resident staffing has a significant impact on common ED efficiency metrics. EDs should consider interventions to mitigate the impact of these expected inefficiencies.

## INTRODUCTION

Resident training is an enormous component of our healthcare system, made up of a tiered structure of roles and responsibilities as learners pursue the profession of medicine. The “July effect” describes the period in which new interns begin learning patient care while senior residents take on additional responsibility and autonomy at teaching hospitals across the country.^1^ Within the medical community it has long been assumed that the yearly influx of new resident physicians temporarily decreases hospital efficiency, and may contribute to hospital crowding, medical errors and increased wait times for patients.^1^–^8^ Despite these concerning findings, some studies have argued against the “July effect” as a clinically significant entity. Several studies found no change in morbidity and mortality during resident turnover in the fields of surgery and obstetrics.^9^,^10^ At times the media, e.g, an essay “It’s July, the Greenest Month in Hospitals, No Need to Panic” published in the *New York Times*, have attempted to overtly refute the July phenomenon and reassure the community.^1^

The “July effect,” though, may have particular significance in the emergency department (ED). According to the National Hospital Ambulatory Medical Care Survey, there were over 21 million ED visits at teaching hospitals in the United States (U.S.) during 2010.^11^ Annual turnover of resident physicians may temporarily decrease efficiency in the ED during months in which residents have limited experience. Despite this significance, few studies have been done on this topic in the ED setting. Riguzzi and colleagues identified no difference in overall length of stay (LOS) in the ED when comparing months of the academic year.^12^ However, this study evaluated only one parameter, which was likely influenced by multiple contributing factors. Additional variables that may contribute to quality of care in the ED are more closely tied to physician work. These factors include faculty and resident productivity, door-to-doctor time, and time to disposition. The objective of this study was to evaluate the impact of annual resident turnover on these efficiency parameters within the ED in a teaching hospital. We aimed to further clarify the influence of the “July effect” on ED efficiency, and potentially highlight staffing adjustments that may be necessary to improve quality of care in this time period.

## MATERIALS AND METHODS

### Study Design

This was an institutional review board-approved retrospective chart review. We extracted data from the electronic health record (EHR) that spanned two academic years (July 2011 – June 2012, and July 2012 – June 2013), with specific attention to charts from May–August in both years. We defined house staff experience by the month of academic year during which the patient received care. July and August were grouped into the period of least experience (PLE) for each year, while May and June were grouped into the period of most experience (PME) for each year. We analyzed and compared data from the PLE and PME intervals.

### Study Setting and Population

The study included patients evaluated by ED residents who were in post-graduate years (PGY) 1–3 and ED attending physicians at a suburban academic Level I trauma ED with annual volumes of approximately 120,000 visits. Only the initial event for each visit was selected for inclusion. We excluded patients seen by residents in other specialties. Table 1 illustrates the staffing comparing PLE with PME for PGY 1–3 ED residents, ED faculty, and rotating residents represented by number of full-time equivalents (FTE).

### Measurements

Outcomes assessed included the following: door-to-doctor time, as measured by the time from ED presentation until the first recorded assignment of a doctor (attending or resident) to the patient; time to disposition as measured by the time from ED presentation until the first recorded disposition time; and physician productivity as defined by average number of patients per month by faculty and resident personnel. Statistics for each variable of interest were provided for combined year 1 (from July 2011–June 2012) and year 2 (from July 2012–June 2013), for all physicians collectively, resident physician groups only, and attending physician groups only.

**Population Health Research Capsule**

What do we already know about this issue?

*Within the medical community, it has long been assumed that the yearly influx of new resident physicians temporarily decreases hospital efficiency, and may contribute to hospital crowding, medical errors, and increased wait times for patients*.

What was the research question?

*The purpose of this study was to evaluate the impact of annual resident turnover on efficiency parameters within the emergency department (ED) in a teaching hospital*.

What was the major finding of the study?

*The annual resident turnover impacts physician productivity, door-to-doctor time, and time-to-disposition in the ED with inefficiencies highlighted during the early segment of the academic year*.

How does this improve population health?

*Resident training is an enormous component of our healthcare system. The annual resident turnover appears to be a clinically relevant factor in the quality and efficiency of patient care. Teaching facilities should consider interventions to mitigate the impact of these expected inefficiencies*.

### Data Analysis

We calculated means, standard deviations (SD) and medians for each outcome variable. Percent change from the PLE to PME was provided for the total number of patients seen and all time variables deemed statistically significant. We constructed mixed-effects models treating “time of year” as a fixed effect and “resident” as a random effect. We performed standard model diagnostics and conducted F-tests of significance for the fixed effects, using the Satterthwaite approximation for denominator degrees of freedom. We performed analysis using R software version 3.1.0. A p-value of less than 0.05 was deemed significant.

## RESULTS

Between July 2011 and June 2013, 79,921 patient records were reviewed. Compared to 40,399 patients in the PME, 39,522 patients were seen in the PLE. Each patient in the study was assigned to one of 190 doctors: 73 attending physicians and 117 emergency medicine residents out of 152 total residents (77%). Patients seen by 35 residents from other specialties were excluded. The number of ED visits by time of year is displayed in Table 2.

There is a slight increase in total patient volume throughout the two-year study. We were unable to detect a significant difference in patient volume between the beginning and end of the academic year (p=0.45). Table 2 illustrates the number of patients seen by type of doctor – resident or attending – during each period of interest. Patients were evaluated by attending physicians alone or in resident/attending combination. For those patients who were evaluated by a resident, it is assumed that the resident was the first doctor to see the patient. There was no difference in mortality between groups (p=0.7652).

### Physician Productivity

Attending physicians saw 10.3% fewer cases primarily at the end of the year than at the beginning of the year (p<0.001). Resident physicians saw 15.8% more patients toward the end of the year compared to the beginning (p<0.0001). Table 3 illustrates these results.

### Door-to-doctor Time

**Figure 1:**
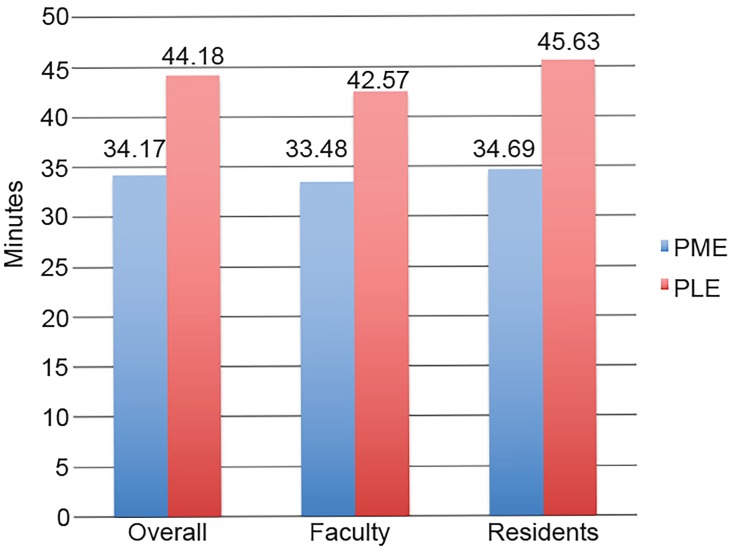
Door to doctor interval for residents, faculty, and all physicians. *PLE*, period of least experience (July/August); *PME*, period of most experience (May/June).

The average door-to-doctor time during the PLE was 44.18 minutes (SD 32.89, median 35) compared to 34.17 minutes (SD 24.9, median 27) during the PME for all practitioners. The residents’ average door-to-doctor time during the PLE was 45.63 minutes (SD 33.01, median 36) compared to 34.69 minutes (SD 25.22, median 28) during the PME, with a significant decrease in time by 21.3% (p=0.0203). The attendings’ average door-to- doctor time during the PLE was 42.57 minutes (SD 32.67, median 34) compared to 33.48 minutes (SD 24.64, median 27) during the PME, with a significant decrease in time by 14% (p<0.0001). Figure 1 graphically illustrates the average door-to-doctor time for providers during the PLE and PME.

### Time to Disposition

**Figure 2:**
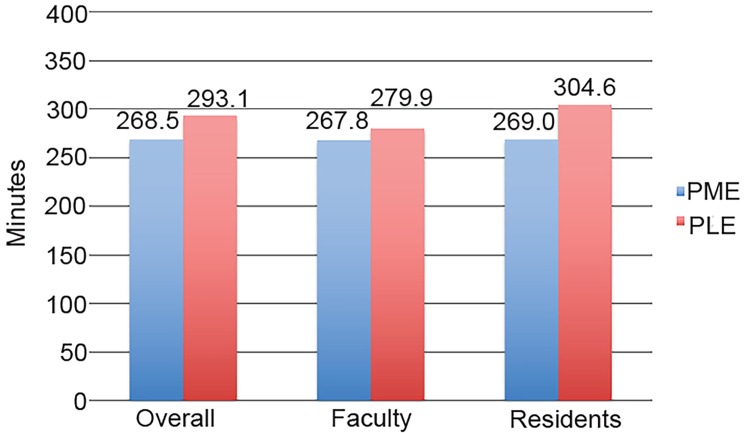
Time to disposition interval for residents, faculty, and all physicians. *PLE*, period of least experience (July/August); *PME*, period of most experience (May/June).

The average time to disposition during the PLE was 293.1 minutes (SD 319.5, median 204) compared to 268.5 minutes (SD 326.5, median 186) during the PME for all physicians. The residents’ average time to disposition during the PLE was 304.6 minutes (SD 308, median 217) compared to 269.0 minutes (SD 282, median 194) during the PME, decreasing the time throughout the year by 12.4% (p=0.0001) (95% confidence interval [2.5% to 23.0%]) The attendings’ average time to disposition during the PLE was 279.9 minutes (SD 331.5, median 186) compared to 267.8 minutes (SD 378.9, median 175) during the PME, which was not a significant change between the beginning and end of the year (p=0.3713). Figure 2 graphically illustrates the average time to disposition for providers during the PLE and PME.

## DISCUSSION

Teaching hospitals are the training grounds for more than 100,000 new practitioners in the U.S. each year and provide care to millions of patients.^13^ As new interns begin learning patient care and senior residents take on additional responsibility and autonomy at teaching hospitals across the country each July, the question remains whether this progression impacts efficiency and patient care. The topic has come to the forefront in mainstream media, and is on patients’ minds as they perceive the healthcare quality they receive.^1^ While there are limited publications in terms of the efficiency impact of the “July effect” across medical specialties, this topic is very relevant to emergency care at teaching facilities and requires further inquiry.

Tracking efficiency metrics is relevant as there is a strong link between quality of care and efficiency. Specifically, early patient contact with an emergency provider is linked to improved quality. In a very large analysis of emergency visits in Australia with nearly six million patients, researchers found that a rapid assessment and triage improved ED length of stay (LOS), ED mortality, and elective-inpatient mortality.^14^ Although we did not find a difference in mortality in our study, it is likely that the sample size was underpowered for this outcome. In another evaluation of 2,619 hospitals, it was evident that each additional hour of ED LOS was associated with a 0.7% decrease in top satisfaction rating and reduction in “definitely recommend the hospital.” A one-hour increase in ED LOS was associated with a 44% increase in the odds that the patient would leave without being seen (LWBS).

Applying overall ED LOS as an efficiency metric has some limitations. Riguzzi et al. found that ED LOS does not differ by month of the academic year in a teaching hospital; rather, it is steadily slower throughout the year when compared to non-teaching hospitals. We found that other parameters of efficiency do in fact differ. We specifically did not use ED LOS as an outcome measure since this outcome is heavily influenced by many variables outside the control of the physician such as hospital occupancy, ED admissions, and number of elective surgical cases.^15^ Instead, the time intervals chosen for this study included door-to-doctor time and time to disposition – two time intervals that the physician more directly influences. While it is intuitive and expected that increasing experience over the course of the academic year improves efficiency metrics, there is minimal existing literature that quantifies this change in efficiency. Our goal was to fill this void and provide baseline guidance.

We found that residents had a longer door-to-doctor time and time to disposition at the beginning of the academic year. Further, resident-physician productivity increased substantially over the course of the academic year. While the results are statistically significant, the clinical impact is more difficult to measure. On average, the door-to-doctor time and time to disposition improved from the PLE to PME by 10 minutes and 35 minutes, respectively. Further elucidation is necessary to determine whether these time parameters make a tangible impact on quality of care and patient satisfaction. One study evaluating door-to-room times and the impact on LWBS rates found a goal rate of less than 1% could be met in patients waiting less than 20 minutes. When patients waited between 21 and 35 minutes, the likelihood of meeting the LWBS goal dropped by 74%. Small changes in efficiency metrics can have a meaningful impact on patient care.^16^

Furthermore, efficiency metrics are inherently intertwined with modification of one variable potentially impacting other variables. Applying these trends may support the introduction of additional interventions to improve efficiency metrics during transition periods. Successful interventions could target provider staffing, ancillary staffing, or other diagnostic testing. Further prospective studies are needed to evaluate this notion because staffing EDs can be complex and costly.

## LIMITATIONS

This study had some limitations, including its retrospective nature. Additional factors may have influenced the outcomes measures. We considered potential covariates in our analysis and made points to account for these. For instance, we confirmed that there was no additional staffing at this hospital during the resident entry month of July. By comparing attending and resident physicians jointly and independently, we considered resident performance separately and resident influence on attending’s patient care performance throughout the academic year. However, there are a number of variables impacting LOS that cannot be quantified. These variables include transport delays, equipment malfunction, information technology upgrades and mishaps, and patient flow within the hospital.

The use of the electronic health record to capture relevant time intervals has some inherent margin of error. For instance, the time-to-disposition interval represents the time from when the patient is evaluated by the provider until a, disposition decision has been made. It is possible that the time stamp when the physician signed up to see the patient is not when the patient was actually evaluated. Finally, although the volume of patients was similar between the PLE and PME, we did not specifically evaluate for differences in overall acuity. It is possible that patients seen at the beginning of the year had a higher acuity that those patients at the end of the year and the complexity of the cases created inefficiencies. Further, while staffing was similar between the PLE and PME, minor differences in FTE per level of training may have impacted the efficiency parameters.

## CONCLUSION

This study shows that resident training impacts several efficiency metrics for patient care with increasing experience related to better performance. The annual resident turnover appears to be a clinically relevant factor in the quality and efficiency of patient care at this teaching hospital. EDs should consider interventions to mitigate the impact of these expected inefficiencies. Further investigations are needed to evaluate any targeted intervention.

## Figures and Tables

**Table 1:** Staffing comparison from period of least experience (PLE) to period of most experience (PME) represented as average full-time equivalents per month.

	PLE (July/August)	PME (May/June)
PGY 1	6.13	6.43
PGY 2	6.43	5.44
PGY 3	11.25	10.5
Rotating resident	3.5	5.0
Attending physician	34.74	35.17
Mid level provider	4.4	4.4
Overall	66.45	62.94

*PGY*, postgraduate year.

**Table 2:** Number of emergency department patient visits by time of year.

	Number of patient visits	PME (May/June)	Number of patient visits
Year 1 (July 2011 – June 2012) n= 39,268			
PLE (July/August)			
July 2011	9,678	May 2012	10,193
August 2011	9,748	June 2012	9,649
Total	19,426		19,842
Year 2 (July 2012 - June 2013) n= 40,653			
PLE (July/August)			
July 2012	9,974	May 2013	10,599
August 2012	10,122	June 2013	9,958
Total	20,096		20,557

*PLE*, period of least experience; *PME*, period of most experience.

**Table 3:** Number of patients seen per month stratified into attending alone and resident/attending combination.

	Time of year	Mean	SD	Median
Year 1 (July 2011–June 2012)				
Doctor				
Attending	PLE	150.1	83.55	149
Attending	PME	136.1	68.84	129
Resident	PLE	188.2	107.88	167
Resident	PME	201.2	96.59	168
Year 2 (July 2012–June 2013)				
Doctor				
Attending	PLE	149.8	77.24	147
Attending	PME	136.9	72.22	123
Resident	PLE	203.9	96.21	187
Resident	PME	209.4	103.17	192

*PLE*, period of least experience; *PME*, period of most experience; *SD*, standard deviation.
